# Chicken miR-148a-3p regulates immune responses against AIV by targeting the MAPK signalling pathway and IFN-γ

**DOI:** 10.1186/s13567-023-01240-3

**Published:** 2023-11-22

**Authors:** Thi Hao Vu, Yeojin Hong, Jubi Heo, Suyeon Kang, Hyun S. Lillehoj, Yeong Ho Hong

**Affiliations:** 1 Department of Animal Science and Technology, Chung-Ang University, Anseong, 17546 Republic of Korea; 2 Animal Biosciences and Biotechnology Laboratory, Agricultural Research Services, United States Department of Agriculture, Beltsville, MD 20705 USA; 3 Department of Biochemistry and Immunology, National Institute of Veterinary Research, Hanoi, 100000 Vietnam

**Keywords:** miR-148a, chicken, MAPK signalling pathway, IFN-γ, HPAIV, H5N1

## Abstract

MicroRNAs are involved in the immune systems of host animals and play essential roles in several immune-related pathways. In the current study, we investigated the systemic biological function of the chicken miRNA gga-miR-148a-3p on immune responses in chicken lines resistant and susceptible to HPAIV-H5N1. We found that gga-miR-148a expression in the lung tissue of H5N1-resistant chickens was significantly downregulated during HPAIV-H5N1 infection. Overexpression of gga-miR-148a and a reporter construct with wild type or mutant IFN-γ, MAPK11, and TGF-β2 3′ untranslated region (3′ UTR)-luciferase in chicken fibroblasts showed that gga-miR-148a acted as a direct translational repressor of IFN-γ, MAPK11, and TGF-β2 by targeting their 3′ UTRs. Furthermore, miR-148a directly and negatively influenced the expression of signalling molecules related to the MAPK signalling pathway, including MAPK11, TGF-β2, and Jun, and regulated antiviral responses through interferon-stimulated genes and MHC class I and class II genes by targeting IFN-γ. Downstream of the MAPK signalling pathway, several proinflammatory cytokines such as IL-1β, IFN-γ, IL-6, TNF-α, IFN-β, and interferon-stimulated genes were downregulated by the overexpression of gga-miR-148a. Our data suggest that gga-miR-148a-3p is an important regulator of the MAPK signalling pathway and antiviral response. These findings improve our understanding of the biological functions of gga-miR-148a-3p, the mechanisms underlying the MAPK signalling pathway, and the antiviral response to HPAIV-H5N1 infection in chickens as well as the role of gga-miR-148a-3p in improving the overall performance of chicken immune responses for breeding disease-resistant chickens.

**Supplementary Information:**

The online version contains supplementary material available at 10.1186/s13567-023-01240-3.

## Introduction

Influenza viruses are single-stranded, segmented, negative-strand RNA viruses [1] of the family *Orthomyxoviridae* consisting of four genera: influenza A, B, and C and *Thogotovirus* [2]. Among those, influenza A viruses are thought to be the only type that triggers natural infections in birds [3]. Avian influenza viruses (AI viruses) cause devastating infections affecting poultry worldwide. However, no efficient control strategies for AI viruses that can be applied in every country and to all kinds of bird species are available [4]. Thus, effective methods for controlling avian influenza viruses, which cause serious economic loss and affect animal welfare, are needed.

MicroRNAs (miRNAs) are small, noncoding RNAs that regulate gene expression by posttranscriptional suppression [5, 6]. Various biological roles of miR-148a-3p have been established among vertebrates. In epithelial ovarian cancer tissues and cell lines, miRNA-148a-3p suppresses the metastasis of ovarian cancer by targeting c-Met [7]. It also functions as a tumour suppressor in epithelial ovarian cancer. In the renal tissues and blood serum of mice and patients, miR-148a-3p promotes proliferation and contributes to lupus nephritis progression by targeting phosphatase and tensin homologue deleted on chromosome ten (PTEN) [8]. Moreover, it has been reported that miR-148a-3p can inhibit the c-Jun and MAPK signalling pathways during hepatitis C virus infection in humans [9, 10]. To date, several miR-148a-3p target genes, such as c-Met, c-Jun, PTEN, and Bcl-2, have been identified in humans [7–9, 11]. One of the pioneering studies confirmed that miR-148a-3p regulates skeletal muscle satellite cell differentiation and apoptosis via the PI3K/AKT signalling pathway by targeting the Meox2 gene [12]. However, its potential roles in the regulation of the immune response in avian species remain largely unexplored.

We previously examined the miRNA transcriptome in the lung tissues of two Vietnamese Ri chicken lines infected with highly pathogenic avian influenza virus (HPAIV)-H5N1 and identified 12 differentially expressed miRNAs between the two lines by high-throughput sequencing. These findings improved the available information on chicken miRNAs, contributed to our understanding of the genetic basis of HPAIV-H5N1 resistance in chickens and provided the basis for a new foundation for novel biomarker identification in an avian model of disease resistance. To investigate the regulatory role of miR-148a, a candidate immune-related miRNA, in avian influenza virus infection, we conducted this study to explore this regulatory role at both the cellular level and the lung tissue level. Our data elucidate the biological and immunological function of gga-miR-148a-3p, validate the differential expression patterns of gga-miR-148a-3p in HPAIV-H5N1 infection-resistant and -susceptible chickens, and identify target genes with critical functions in chicken innate immune responses. This could potentially provide insights into the mechanisms underlying resistance to avian influenza virus infection and contribute to the development of new treatments or prevention strategies.

## Materials and methods

### Animals and sample preparation for next-generation sequencing

The Ri chickens resistant or susceptible to HPAIV-H5N1 infection used in this study were provided by the National Institute of Animal Science (NIAS, Hanoi, Vietnam). For H5N1 infection, 4-week-old mixes of male and female chickens (five chickens per group) were infected intranasally with the harvested allantoic fluid from infected eggs, which contained 1 × 10^4^ 50% egg infectious dose (EID50) of A/duck/Vietnam/QB1207/2012 (H5N1) [13]. Lung tissues were collected 3 days after infection. All animal protocols were approved by the National Institute of Veterinary Research (NIVR), Vietnam (TCVN 8402:2010/TCVN 8400-26:2014). Total RNA from lung tissues of resistant and susceptible Ri chickens was extracted. After the quality check, RNA samples were subjected to small RNA-seq by individual samples by the LAS (Gimpo, South Korea) on an Illumina MGISEQ platform (Illumina Inc., San Diego, CA, USA). Sequencing data analysis was carried out as previously described [13].

Potentially existing sequencing adapters and low-quality bases in reads were trimmed by Skewer ver 0.2.2 [14]. The cleaned high-quality reads after trimming the low-quality bases and sequencing adapters were mapped to the reference genome specifically for miRNAs using QuickMIRSeq [15]. All known mature miRNAs and hairpins were downloaded from miRBase release 22.1, 2019 [16]. To quantify miRNA expression levels from the mapped reads on the reference genome, QuickMIRSeq [15] was used with default options. The miRNA and hairpin annotations from miRBase [16] were used, and the expression values were calculated in Reads Per Million (RPM) units. The differentially expressed miRNAs between the two selected biological conditions (differential expression probability > 0.9) [17] were analysed using NOISeq ver 2.16.0 in Bioconductor. The miRNA gga-miR-148a-3p (MIMAT0001120) was selected for further analyses because it was found to be differentially expressed in HPAIV-infected resistant chickens compared with susceptible chickens (Additional file 1).

### Genes targeted by gga-miR-148a-3p

The miRDB v6.0 [18] chicken miRNA database custom prediction mode was used to predict gga-miR-148a-3p target genes. Of these, genes associated with relevant gene ontology (GO) terms and immune-related pathways, such as the MAPK signalling pathway, were selected using DAVID [19] analysis. Immune-related target genes that scored above 80 were selected for further analysis (Additional file 2).

### RT-qPCR for candidate target genes and miRNAs

The total RNA from resistant and susceptible chicken lines was individually reverse transcribed into cDNA using a RevertAid First Strand cDNA Synthesis Kit (Thermo Fisher Scientific, Waltham, MA, USA) according to the manufacturer’s instructions. Primers were designed using Primer-BLAST [20] (Additional file 3). Quantitative PCR (qPCR) was performed using a Dyne qPCR 2× PreMIX (Dyne Bio, Seongnam, South Korea) solution in 40 cycles of incubation at 95 °C for 30 s, 55–60 °C for 30 s, and 72 °C for 30 s in a CFX Connect Real-time PCR (Bio-Rad, Hercules, CA, USA) instrument according to the manufacturer’s instructions.

To amplify miRNA via RT-qPCR, a miRNA-specific forward primer was designed based on the full length of the mature miRNA sequence, and a guanine nucleotide was added to the 5′ end of the sequence to adjust the Tm (Additional file 3). A universal primer from the Ncode™ miRNA First-Strand cDNA Synthesis Kit (Invitrogen) was used as the reverse primer. The total RNA was reverse-transcribed into cDNA using the NCode miRNA First-Strand cDNA Synthesis Kit (Invitrogen). Quantitative PCR was performed using AMPIGENE® qPCR Green Mix Lo-ROX (Enzo Life Sciences) in 45 cycles of incubation at 95 °C for 15 s and 55 °C for 30 s in a CFX Connect Real-time PCR (Bio-Rad) device according to the manufacturer’s instructions. Threshold cycle (Ct) values were normalized to GAPDH (mRNA) or U1A (miRNA) expression using the 2^−ΔΔCt^ method [21]. All reactions were performed in triplicate.

### Mimic miRNA transfection and poly I:C treatment

The chicken HD11 macrophage line [22] was cultured in RPMI-1640 medium with l-glutamine (Gibco, Grand Island, NY, USA) supplemented with 10% foetal bovine serum (Gibco) and 1% penicillin‒streptomycin (Gibco) at 41 °C (5% CO_2_). The gga-miR-148a-3p precursor sequence was obtained from miRbase [23], and the control RNA duplex (gga-miR-NC; sense strand, UUCUCCGAACGUGUCACGUTT) was nonhomologous to the chicken genome sequence [24]. The gga-miR-NC and gga-miR-148a-3p mimic oligonucleotides were chemically synthesized by Bioneer (Daejeon, South Korea). Four treatment groups were included in the experiment: (i) mimic gga-miR-NC transfection, (ii) mimic gga-miR-NC transfection and high molecule weight polyinosinic-polycytidylic acid (Poly I:C) treatment, (iii) mimic gga-miR-148a-3p transfection, and (iv) mimic gga-miR-148a-3p transfection and Poly I:C treatment. To transfect mimic miRNAs, synthetic gga-miR-NC or gga-miR-148-3p oligonucleotide mimics were transfected in triplicate at a final concentration of 30 nM using Lipofectamine RNAiMAX (Invitrogen) according to the manufacturer’s instructions. Briefly, chicken HD11 macrophages were seeded into a 12-well plate and incubated for 4 h. Then, 30 nM of mimic miRNA was diluted in 50 µL of Opti-MEM (Gibco), and 3 µL of Lipofectamine RNAiMAX was diluted in 50 µL of Opti-MEM for mimic miRNA transfection. After mixing the diluted mimic miRNA and diluted Lipofectamine RNAiMAX together, the mixture was incubated for 5 min at room temperature (23 °C, RT) and added dropwise into the wells. After a 24 h incubation at 41 °C (5% CO_2_), either 5.0 µg/mL of high molecule weight Poly I: C (InvivoGen, Hong Kong) or normal medium was added, and the cells were incubated for 4 h. Total RNA was isolated using TRIzol reagent (Invitrogen) and treated with DNase I (Sigma) to remove genomic DNA contamination.

### Luciferase reporter assay

The wild-type and mutant 3′-UTR DNA fragments of IFN-γ, MAPK11, and TGF-β2 covering the predicted binding sites of gga-miR-148a-3p were successfully cloned. A total of 1.5 × 10^6^ DF1 chicken fibroblasts/well were seeded in a 6-well plate (Corning, NY, USA). Four hours later, cells at 80% confluence were transfected with plasmid DNA using Lipofectamine RNAiMAX (Invitrogen) according to the manufacturer’s recommendations. In brief, the target gene with luciferase vector or mutant target gene with luciferase vector was cotransfected with miR-148a-3p into the cells. The pMIR-REPORT β-gal control plasmid (Ambion, Austin, Texas, USA) was also transfected for normalization. After 24 h, the cells were rinsed with 1× PBS, harvested using a scraper, and lysed in 280 µL of 1× luciferase cell culture lysis reagent (CCLR; Promega, Madison, WI, USA). After vortexing for 10–15 s and centrifuging at 14 000 × *g* for 2 min, the supernatant was used to measure luciferase and β-galactosidase activities in 96-well plates (Corning) in triplicate using Luciferase Assay Systems (Promega) and a β-Galactosidase Enzyme Assay System (Promega), respectively. For the luciferase activity assay, 20 µL/well of the supernatant was mixed with 100 µL/well of Luciferase Assay Reagent in white plates in a dark room, and luciferase activity was immediately measured using a GloMax®-Multi Detection System (Promega). For the β-galactosidase activity assay, 50 µL/well of the supernatant was mixed with 50 µL/well of Assay 2× Buffer in transparent plates followed by incubation at 37 °C for 30 min before the measurement. Reported luciferase activity was normalized to β-galactosidase activity. Each treatment and assay were independently repeated three times.

### Western blot analysis

HD11 chicken macrophages transfected with mimic miRNAs were lysed in Tris-Triton lysis buffer (50 mM Tris-HCl, pH 7.5, 150 mM NaCl, 2 mM EDTA, 100 mM sodium fluoride, 0.1% SDS, 0.5% sodium deoxycholate, 1% Triton X-100, 10 mM sodium pyrophosphate, 10 mM sodium orthovanadate, and 10 mM protease inhibitor). Protein samples were separated by SDS-PAGE on a 10% acrylamide gel and transferred onto a PVDF membrane (Invitrogen). The PVDF membranes were blocked with PBS containing 0.05% Tween 20 (PBST) and 5% nonfat milk and incubated with rabbit anti-chicken phospho-p38 MAPK (Thr180/Tyr182) monoclonal antibody (Cell Signaling Technology, 4631S, cross-reactive, Danvers, Massachusetts, USA) and mouse anti-chicken GAPDH antibody (Thermo Fisher Scientific, AM4300, specific) diluted in PBST containing 2% nonfat milk overnight. The membranes were then washed with PBST. HRP-conjugated anti-rabbit or anti-mouse (Thermo Fisher Scientific) secondary antibodies were used based on the primary antibodies. Protein bands were detected using Western Lightning Plus-ECL substrate (Thermo Fisher Scientific).

### Statistical analysis

All experiments were repeated three times, and the results are expressed as the mean ± standard error of the mean (SEM; *N* = 3). The data were analysed using the statistical software IBM SPSS (SPSS 26.0 for Windows; IBM, Chicago, IL, USA). Differences between groups were analysed by Tukey’s multiple-range method, and those with *p* values lower than 0.05 were considered statistically significant.

## Results

### miR-148a-3p structure and in silico target prediction

**Figure 1:**
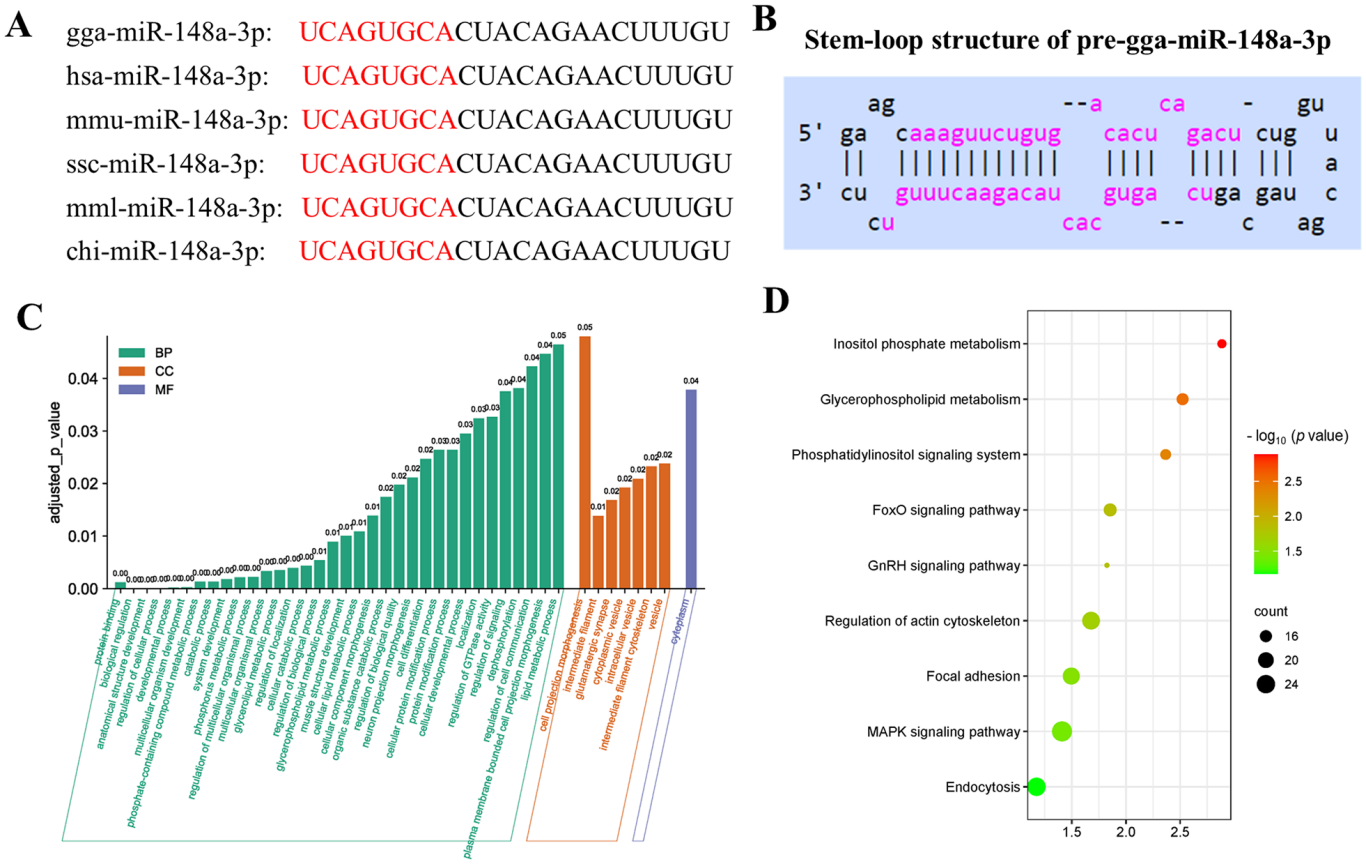
**Sequencing characteristics and biological functions of miR-148a-3p target genes.** **A** Mature sequence (red characters) of miR-148a-3p among different animal species. The miRNA gga-miR-148a-3p is highly conserved across species (gga, *Gallus gallus*; hsa, *Homo sapiens*; mmu, *Mus musculus*; Ssc, *Sus scrofa*; mml, *Macaca mulatta*; Chi: *Capra hircus*). **B** Stem‒loop structure of pre-gga-miR-148a-3p. **C** GO enrichment analysis of 270 target genes. **D** The significantly enriched KEGG pathways associated with 270 target genes.

 We performed sequence alignment analysis and found that the seed sequence of chicken miR-148a-3p (Figure 1A) and the stem‒loop of pre-gga-miR-148a-3p (Figure 1B) were conserved in mammals. Using the miRDB algorithm for miRNA target prediction primarily based on the gga-miR-148a sequence, we found a total of 1200 gga-miR-148a-3p candidate target genes. To verify immune-related genes, GO term and KEGG pathway enrichment analyses were conducted with DAVID and KEGG tools (Figures 1C, D). Of the 27 immune-related candidate target genes (Additional file 2), four genes were selected for further validation as they were associated with immune responses: interferon-gamma (IFN-γ), mitogen-activated protein kinase 11 (MAPK11), TGF-beta activated kinase 1 binding protein 3 (TAB3) and transforming growth factor-beta 2 (TGF-β2). Three of these, MAPK11, TAB3, and TGF-β2, were involved in the MAPK signalling pathway and IFN-γ. To predict the 3′ UTR binding sites of the candidate target genes with gga-miR-148a-3p, the RNA hybrid tool was used based on the seed matches between two molecules: nucleotides 2–8 of the miRNA and the 3′ UTR of the target gene. Binding sites of the potential target genes that met these criteria were selected for further experimental validation.

### The expression of gga-miR-148a-3p and its target genes in the lung tissue of two chicken lines

**Figure 2:**
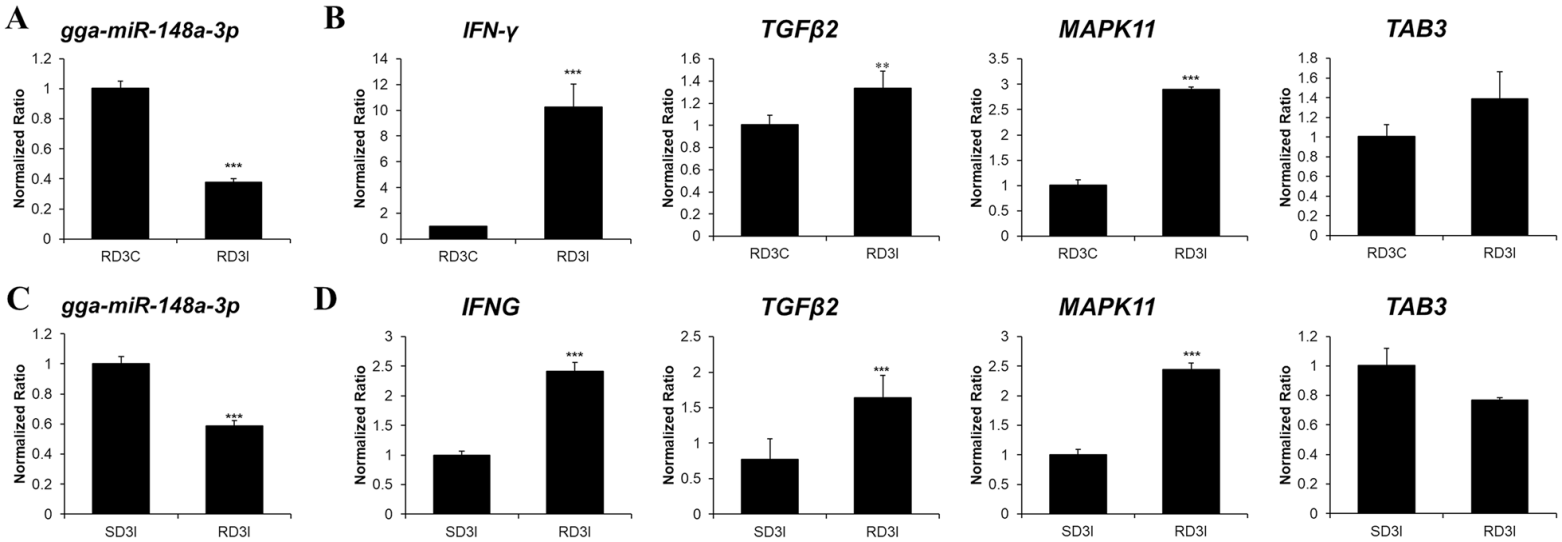
**Expression profiles of gga-miR-148a-3p and its candidate target genes in the lungs of Ri chickens.** **A** The expression levels of miRNA and **B** its target genes in the resistant chickens after H5N1 infection and **C** miRNA and **D** target gene expression in H5N1-infected resistant chickens compared to infected susceptible chickens. For miRNA expression, the RT‒qPCR result was normalized to U1A expression levels. For mRNA, the RT-qPCR result was normalized to glyceraldehyde-3-phosphate dehydrogenase (GAPDH) expression levels. Significant differences in gene expression levels are indicated as follows: *p < 0.05; **p < 0.01; and ***p < 0.001. Error bars indicate the SEM (*n* = 3) of three technical replicates.

The expression of gga-miR-148a-3p was significantly different between control and infected resistant chickens and between infected susceptible and resistant chickens (*p* < 0.01). It was downregulated in H5N1-infected resistant chickens compared to the controls and in H5N1-infected resistant chickens compared to H5N1-infected susceptible chickens (Figures 2A, C). Moreover, the expression levels of the gga-miR-148a-3p candidate target genes IFN-γ, MAPK11 and TGF-β2 were significantly upregulated in H5N1-infected resistant chickens compared to the controls and in the resistant chickens compared to susceptible chickens. However, the expression of the TAB3 gene was not significantly altered after H5N1 infection (Figures 2B, D). The expression levels of MAP2K3, IL17RA, IL18RAP, and IL2RA were significantly upregulated in H5N1-infected resistant chickens compared to the controls and in resistant chickens compared to susceptible chickens (Additional file 4). These results indicated that target gene mRNA expression levels were negatively correlated with gga-miR-148a levels in resistant and susceptible chicken lines.

### Candidate target gene expression upon gga-miR-148a and poly I:C treatment in the HD11 macrophage line

**Figure 3:**
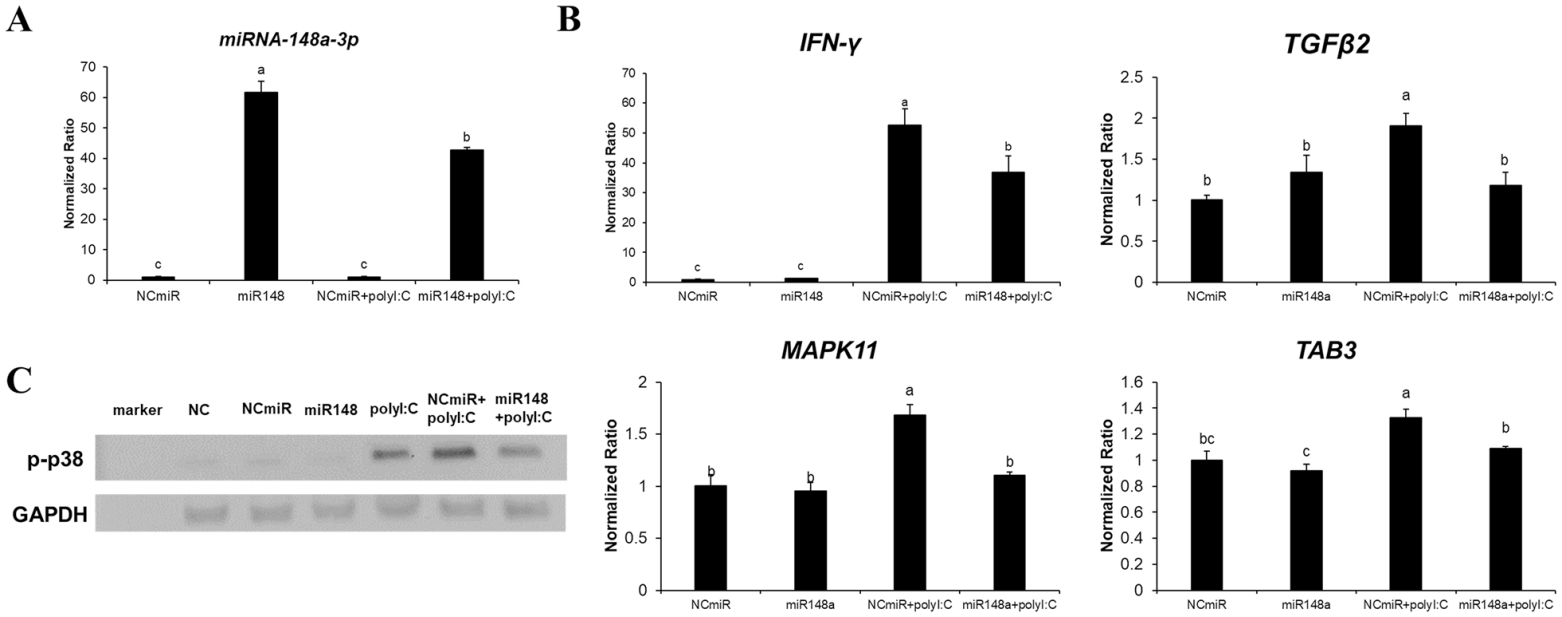
**Chicken mimic miR-148a represses several target genes in the MAPK signalling pathway and antiviral response.** **A** Expression profiles of gga-miR-148a-3p and **B** its candidate target genes in the HD11 chicken macrophage line transfected with mimic miR-148a-3p or NC-miR treated with and Poly I:C. **C** Western blot analysis of p-p38 and GAPDH proteins in the HD11 chicken macrophage line transfected with mimic miR-148a. The levels of miR-148a were analysed by RT-qPCR and normalized to those of endogenous U1A RNA. Gene expression levels were normalized to those of GAPDH. Data are presented as the mean ± SEM (*n* = 3) from three independent experiments. Six treatment groups were included in this experiment: (1) no miRNA, no Poly I:C, (2) mimic gga-miR-NC transfection, (3) mimic gga-miR-148a-3p transfection, (4) Poly I:C treatment, (5) mimic gga-miR-NC transfection and Poly I:C treatment, and (6) mimic gga-miR-148a-3p transfection and Poly I:C treatment. Different lowercase letters above the dots indicate significant differences (*p* < 0.05) among samples as determined by analysis of variance with Tukey’s multiple comparison test.

We investigated whether gga-miR-148a or Poly I:C treatment affected the expression levels of candidate targets of gga-miR-148a. The expression levels of gga-miR-148a-3p and four candidate target genes (IFN-γ, MAPK11, TGF-β2, and TAB3) with or without Poly I:C in HD11 cells are shown in Figure 3. The results showed that the expression levels of gga-miR-148a-3p in mimic miR-148-transfected cells with or without Poly I:C significantly increased (*p* < 0.001) compared to mimic NC-miR-transfected cells treated with or without Poly I:C (Figure 3A). Comparison of IFN-γ, MAPK11, TGF-β2, and TAB3 mRNA expression levels between Poly I:C-treated cells transfected with mimic miR-148a or mimic NC-miR revealed that Poly I:C stimulated IFN-γ, MAPK11, and TGF-β2 gene expression in HD11 cells (Figure 3B). Moreover, the presence of mimic miR-148a significantly repressed IFN-γ, MAPK11, and TGF-β2 expression following Poly I:C treatment (Figure 3B). The translational levels of p-p38 (translational expression of MAPK11 genes) were downregulated in mimic miR-148a-transfected HD11 cells (Figure 3C). These results indicated that gga-miR-148a inhibited IFN-γ, MAPK11 and TGF-β2 expression.

### In vitro validation of the gga-miR-148a target genes IFN-γ, MAPK11, and TGF-β2 in DF1 cells

**Figure 4:**
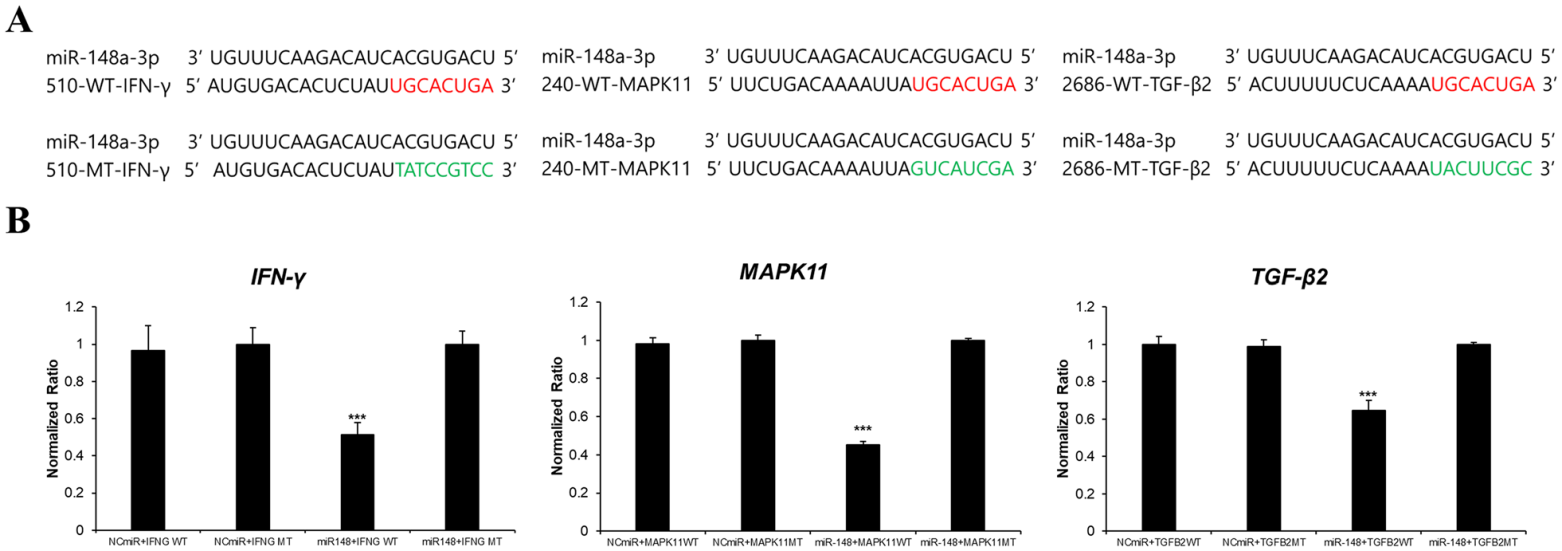
**Luciferase reporter assay**. **A** Schematic diagram of miRNA binding sites and wild-type and mutant target genes (IFN-γ/Luc, Mut-IFN-γ/Luc, MAPK11/Luc, Mut-MAPK11/Luc, TGF-β2/Luc, and Mut-TGF-β2/Luc). The 3′-UTRs of wild-type and mutant target genes were cloned and inserted into a pMIR-REPORT luciferase vector. **B** Luciferase reporter assay performed at 24 h following cotransfection of chicken embryo fibroblast line (DF1) with the wild type or mutant vectors together with mimic miR-148a-3p or NC-miR as control. The results (mean ± SEM) are representative of three independent experiments (*n* = 3). Different lowercase letters above the dots indicate significant differences (*p* < 0.05) among samples as determined by analysis of variance with Tukey’s multiple comparison test.

The luciferase assay was performed in chicken embryo fibroblast DF1 cells cotransfected with wild type (IFN-γ/Luc, MAPK11/Luc and TGF-β2/Luc) or mutant (Mut-IFN-γ/Luc, Mut-MAPK11/Luc and Mut-TGF-β2/Luc) plasmids (Figures 4A, B) and the mimic gga-miR-148a-3p or empty luciferase vector as control. Luciferase activity was significantly reduced in DF1 cells when the mimic gga-miR-148a-3p was cotransfected with a target gene luciferase vector containing a miR-148a binding sequence. However, no differences were observed when these cells were transfected with mutant target gene luciferase vectors (Figure 4B). These results revealed that gga-miR-148a may directly target the chicken IFN-γ, MAPK11, and TGF-β2 genes.

### Gga-miR-148a-3p inhibits the MAPK signalling pathway

**Figure 5:**
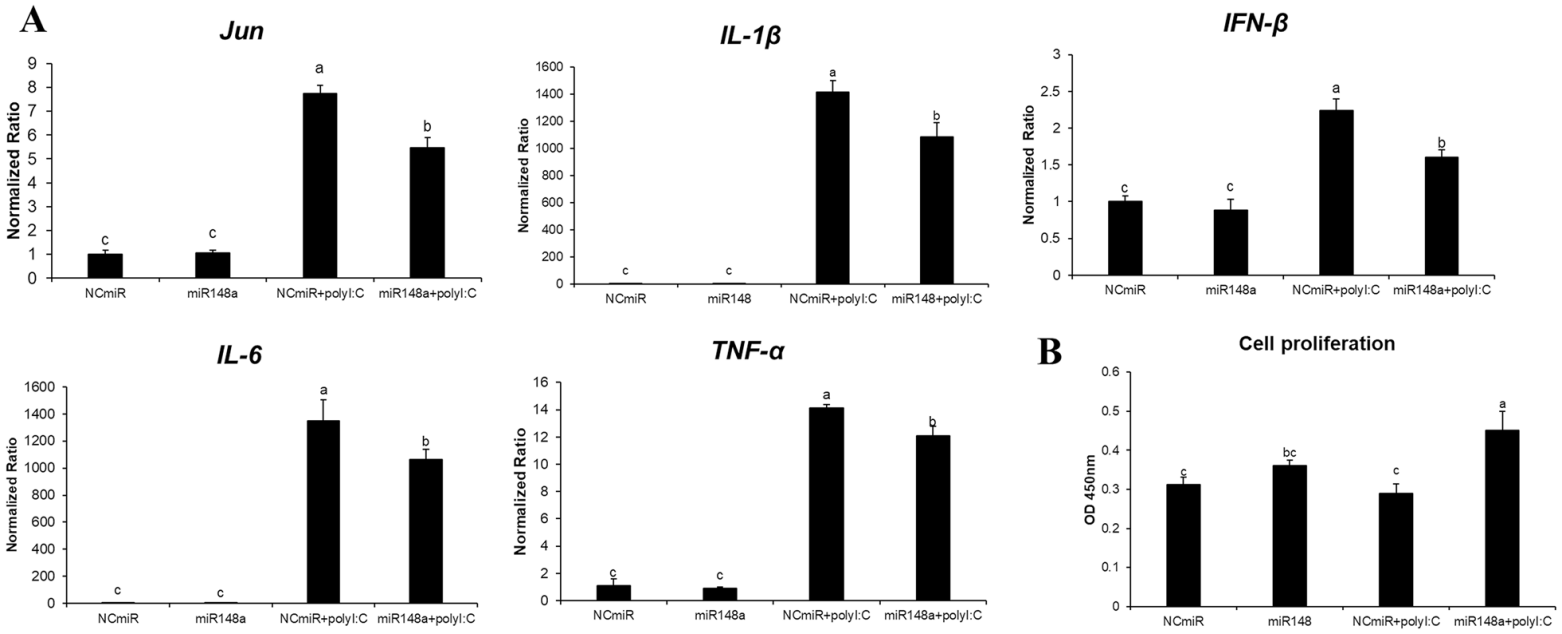
**Cytokine gene expression and cell proliferation.** **A** Expression of cytokines involved in the MAPK signalling pathway and **B** cell proliferation in HD11 chicken macrophages treated with mimic miRNA and treated with Poly I:C. Gene expression levels were normalized to those of GAPDH. Data are presented as the mean ± SEM (*n* = 3) from three independent experiments. Different lowercase letters above the dots indicate significant differences (*p* < 0.05) among samples as determined by analysis of variance with Tukey’s multiple comparison test.

The transcriptional levels of MAPK signalling molecules were downregulated in cells expressing the mimic miRNA (Figure 3). That is, miR-148a inhibited the expression of MAPK11, TGF-β2, TAB3, and Jun after Poly I:C treatment (Figures 3 and 5). In addition, in vivo results also showed that the expression levels of MAPK11 and TGF-β2 mRNA were elevated in resistant chickens after AIV infection and in resistant chickens compared to susceptible chickens (Figures 2B, D).

The expression profiles of cytokines involved in the MAPK signalling pathway were also evaluated. In resistant chickens, the mRNA levels of IFN-γ, IFN-β, IL-1β, IL-6, and IL-8 were significantly higher in H5N1-infected lung samples and in resistant chickens than in susceptible chickens [13]. The cytokine expression patterns in the mimic miR-148a-transfected cells with or without Poly I:C treatment revealed that IFN-γ, IL-1β, IFN-β, IL-6, and TNF-α expression levels were negatively correlated with the combination of gga-miR-148a expression and Poly I:C treatment (Figures 3B and 5). Specifically, the expression levels of IFN-γ, IFN-β, IL-1β, IL-6, and TNF-α increased 65-fold, 3.5-fold, 1500-fold, 1500-fold, and 15-fold upon Poly I:C treatment, respectively, and were significantly repressed by mimic miR-148a (Figure 5A). The expression levels of IFN-γ, IFN-β, IL-1β, IL-6, and TNF-α located downstream of the MAPK signalling pathway are directly regulated as a result of MAPK11 and TGF-β2 regulation by miR-148a.

The MAPK signalling pathway also regulates cell proliferation. Therefore, we detected cell proliferation after miRNA transfection and Poly I:C treatment. The cell proliferation rate increased upon miR-148 mimic transfection and Poly I:C treatment (Figure 5B). These results indicate that mimic-148a may negatively regulate the MAPK signalling pathway and suppress the expression of proinflammatory cytokines in the HD11 chicken macrophages.

### The miRNA gga-miR-148a-3p inhibits the expression of IFN-γ, which induces interferon-stimulated genes and major histocompatibility complex (MHC) class genes

**Figure 6:**
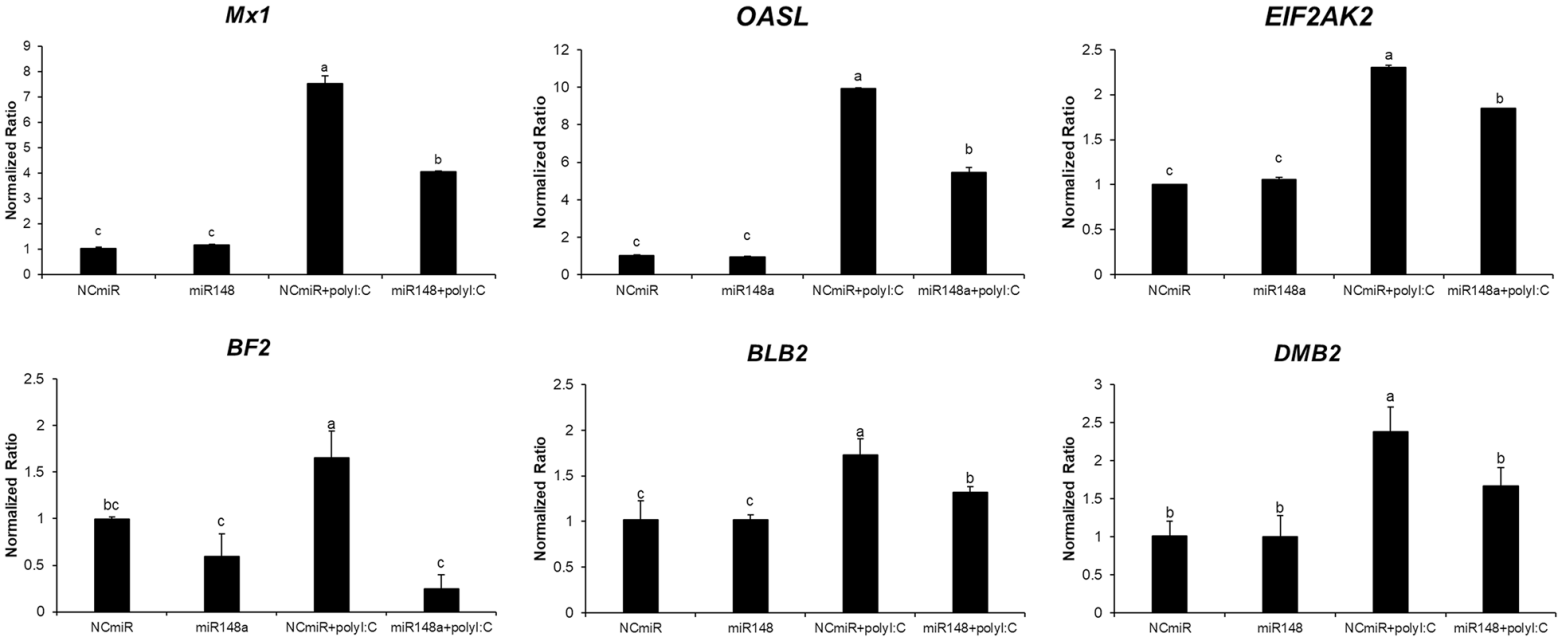
**Expression levels of interferon-stimulated genes and MHC class genes in HD11 chicken macrophages transfected with mimic miRNA and treated with Poly I:C.** Gene expression levels were normalized to those of GAPDH. Data are presented as the mean ± SEM (*n* = 3) from three independent experiments.

The transcriptional levels of interferon-stimulated genes (Mx1, OASL and EIF2AK2) were downregulated upon mimic miRNA transfection followed by Poly I:C treatment. Of these, the mRNA levels of Mx1, OASL, and EIF2AK2 were downregulated eightfold, tenfold, and 2.3-fold, respectively, by mimic miR-148a transfection (Figure 6).

Moreover, the transcript levels of MHC class I (BF2) and class II (BLB2 and DMB2) signalling molecules were downregulated in cells transfected with the mimic miRNA and treated with Poly I:C (Figure 6). Investigation of the MHC class expression patterns in the mimic miR-148a-transfected cells with or without Poly I:C treatment revealed that the levels of BF2, BLB2, and DMB2 were negatively correlated with the combination of gga-miR-148a expression and Poly I:C treatment. Of these, the transcript levels of BF2, BLB2, and DMB2 increased twofold, twofold, and threefold upon Poly I:C treatment, respectively, and were significantly repressed by mimic miR-148a (Figure 6). In brief, it is possible that the expression levels of interferon-stimulated genes and the MHC class genes involved in antiviral activity are indirectly regulated in chickens as a result of IFN-γ regulation by miR-148a.

## Discussion

An increasing number of studies are being conducted on the crucial roles of microRNAs, such as gga-miR-200a-3p [25] and gga-miR-10a [26], in mediating the innate host responses of chickens against pathogens. In this study, the biological function of gga-miR-148a, especially in host responses to HPAIV-H5N1 infection in Vietnamese native chickens, was investigated.

The mitogen-activated protein kinase (MAPK) signalling pathway plays an essential role in innate immunity and is involved in several cellular functions, such as inflammation, cell proliferation, cell differentiation, and cell death [27]. Previous studies have shown that the influenza virus activates MAPK pathway genes, including p38 MAPK, and this change further increases the expression of cytokines or affects the proliferation of viruses [28]. In addition, in vitro and in vivo studies have shown that inhibition of the MAPK signalling pathway can protect mice against H5N1 [29, 30]. In our previous study using the same animal model, i.e., Ri-resistant and Ri-susceptible chickens, we found that the expression of genes involved in the cytokine‒cytokine receptor signalling pathway, MAPK signalling pathway, and influenza A pathway was significantly altered in H5N1-infected chickens; these pathways are important in innate immune defence and foreign DNA sensing [13, 31–33]. In the HPAIV-H5N1-infected chicken model, miR-148a expression was reduced in the resistant chickens, which showed resistance to H5N1. In contrast, the expression of genes predicted to be gga-miR-148a-3p targets (MAPK11 and TGF-β2), which are involved in the MAPK pathway, was upregulated in response to H5N1 infection. Upon miR-148a mimic transfection, the expression levels of MAPK11 and TGF-β2 were significantly downregulated in chicken macrophages, which resulted in a decrease in the levels of the downstream signalling molecule Jun. After Poly I:C stimulation, the upregulation of MAPK11 and TGF-β2 was followed by increases in the levels of proinflammatory cytokines (IL-1β, IFN-γ, IL-6, TNF-α, and IFN-β), which were markedly suppressed by miR-148a overexpression, suggesting that gga-miR-148a may be involved in the regulation of the MAPK signalling pathway. Moreover, the luciferase reporter assay confirmed MAPK11 and TGF-β2 as direct targets of miR-148a through the expected binding of the 3′-UTRs of MAPK11 and TGF-β2 with miR-148a. In addition, gga-miR-148a acts as a direct translational repressor of p38 (MAPK11), as confirmed by Western blotting. These results are similar to those of previous studies on the biological activities of miR-148a-3p in humans and mice through the MAPK signalling pathway [7–9]. Taken together, these results suggest that gga-miR-148a is an inhibitor that targets the MAPK signalling pathway directly through the MAPK11 and TGF-β2 genes.

IFN-γ activates T helper 1-type immune responses [34]. Furthermore, several studies have defined the protective role IFN-γ against viruses such as Newcastle disease virus, Marek’s disease virus, and influenza viruses [35–37]. The biological activity of IFN-γ is induced by stimulating the expression of class I and class II MHCs, which play important roles in the adaptive immune system through the JAK/STAT1 signal transduction pathway [38, 39]. Both the in vivo and in vitro results of this study confirmed IFN-γ as a direct target of miR-148a. Moreover, the expression levels of MHC class I (BF2) and class II (DMB2 and BLB2) were significantly downregulated after miR-148a transfection and Poly I:C treatment compared to the controls. IFN-γ has been reported to tightly regulate nitric oxide production, which is an important cellular inhibitory mechanism against virus-induced myocarditis [40]. However, other studies have shown that interferon-γ did not induce any detectable increases in nitric oxide production [41]. Our results indicated that nitric oxide production is not detectable after miR-148a transfection and Poly I:C treatment in chicken macrophages (data not shown). Chicken IFN-γ displays antiviral activities in vitro [42] and induces the upregulation of some interferon-stimulated genes, such as 2′–5′ OAS, against influenza A virus [43, 44]. IFN-stimulated genes can inhibit viral replication by blocking virus entry into host cells, binding to viral RNA to block translation, and regulating host antiviral responses [45]. In our study, interferon-stimulated genes (Mx1, OASL and EIF2AK2) were significantly downregulated in miR-148a-transfected cells treated with Poly I:C compared to the controls. Taken together, these results indicate that it is possible that gga-miR-148a regulates antiviral responses through interferon-stimulated genes and MHC class genes by targeting the IFN-γ gene.

**Figure 7:**
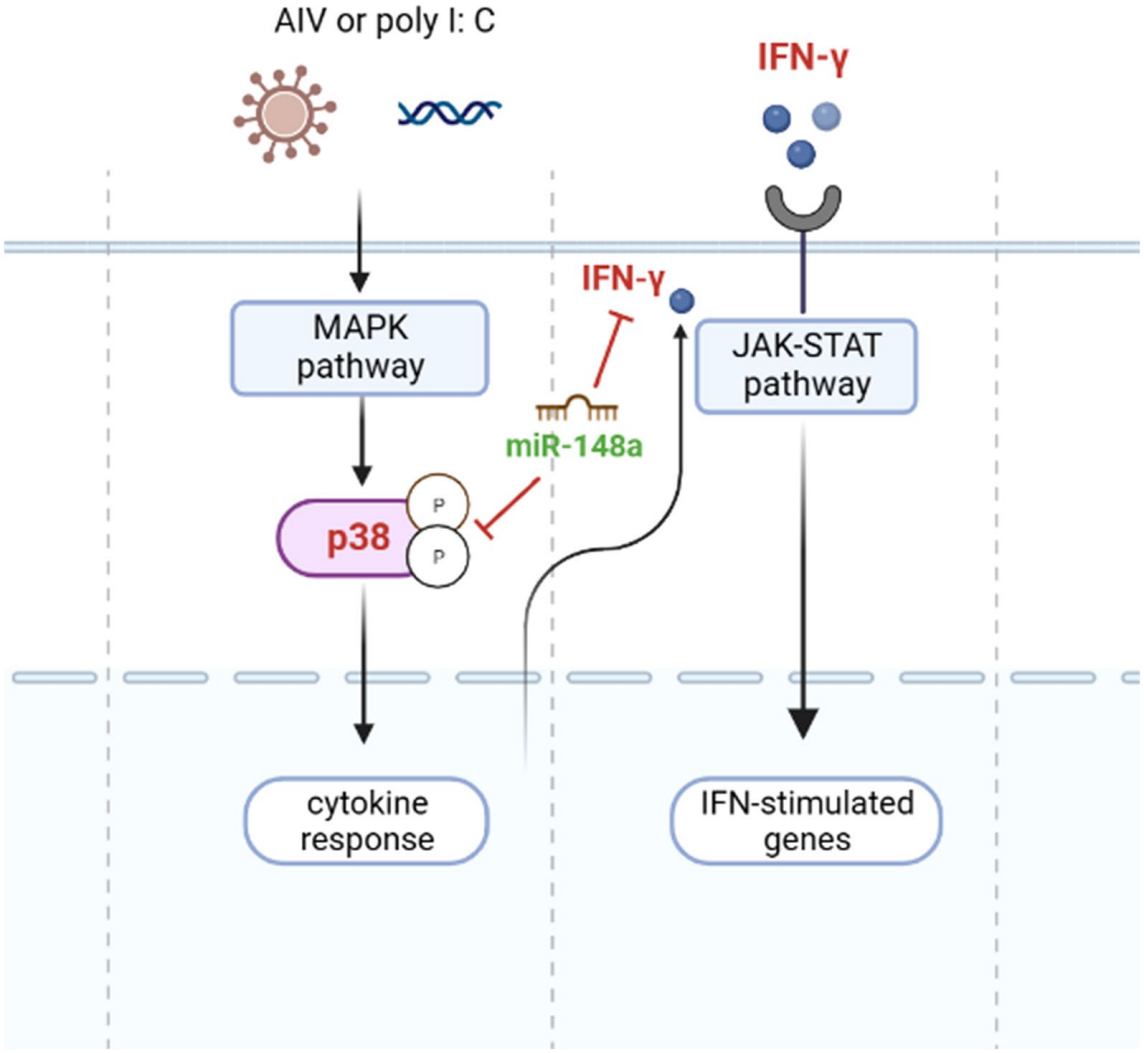
**Summary demonstrating the potential function of gga-miR-148a as an inhibitor of the MAPK signalling pathway and antiviral response.**

In conclusion, miR-148a regulates the immune response in chickens by targeting the IFN-γ, MAPK11, and TGF-β2 genes. Downregulation of miR-148a in an HPAIV-H5N1-infected chicken model may induce an innate immune response through the MAPK signalling pathway and promote an antiviral response through the upregulation of IFN-γ, MAPK11, and TGF-β2 (Figure 7). These findings provide valuable insights into the biological functions of miR-148a, the mechanisms of the MAPK signalling pathway, and antiviral responses upon H5N1 infection in chickens and improve the overall understanding of immune responses for breeding disease-resistant chickens. This study was conducted using local chickens and chicken cell lines, so further studies on commercial chickens are needed.

### Supplementary Information

**Additional file 1. List of differentially expressed miRNAs in the resistant and susceptible lines, as observed at 3 days postinfection.**

**Additional file 2. List of immune-related target genes scoring over 80 in the miRDB.**

**Additional file 3. Sequences of primers used for quantitative PCR or cloning.**

**Additional file 4. The gene expression of immune-related target genes (in the miRDB database) of gga-miR-148a-3p in the lung tissues of resistant chickens after H5N1 infection (A) and in H5N1-infected resistant chickens compared to H5N1-infected susceptible chickens (B).** Expression levels were normalized to those of GAPDH and measured in triplicate. Significant differences in mRNA expression levels are indicated as follows: *, *p* < 0.05; **, *p* < 0.01; and ***, *p* < 0.001. Error bars indicate the SEM of technical replicates performed in triplicate.

## Data Availability

All data generated or analysed during this study are included in this published article and its additional information files.

## Abbreviations

MAPK: mitogen-activated protein kinase
HPAIV: highly pathogenic avian influenza virus
KEGG: Kyoto Encyclopedia of Genes and Genomes
IL: interleukin
IFNs: interferons
TGF: transforming growth factor
Poly I:C: polyinosinic-polycytidylic acid
MHC class: major histocompatibility complex class
