# Supplementary material for: Chicken miR-148a-3p regulates immune responses against AIV by targeting the MAPK signalling pathway and IFN-γ
Source: Vet Res. 2023 Nov 22;54:110. doi: 10.1186/s13567-023-01240-3 (PMC10664352; doi:10.1186/s13567-023-01240-3)
Supplement: Supplementary file 1 — Additional file 1. List of differentially expressed miRNAs in the resistant and susceptible lines, as observed at 3 days postinfection. [file 13567_2023_1240_MOESM1_ESM.docx]

**Additional file 1**. **List of differentially expressed miRNAs in the resistant and susceptible lines, as observed at 3 days post-infection.**

| **miRNA** | **RD3I** | **SD3I** | **Theta** | **Probability** | **log_2_(FC)** | **Significant** |
| --- | --- | --- | --- | --- | --- | --- |
| gga-miR-100-5p | 15323.972 | 6707.02 | 1.653 | 1 | -1.192 | Yes |
| gga-miR-126-5p | 18550.825 | 33036.52 | -1.543 | 1 | 0.833 | Yes |
| gga-miR-140-3p | 3206.347 | 8316.88 | -1.373 | 0.991 | 1.375 | Yes |
| gga-miR-148a-3p | 25594.245 | 41690.29 | -2.428 | 1 | 0.704 | Yes |
| gga-miR-22-3p | 14901.062 | 27068.91 | -1.253 | 0.92 | 0.861 | Yes |
| gga-miR-27b-3p | 5900.398 | 10849.835 | -1.493 | 1 | 0.879 | Yes |
| gga-miR-2954 | 1554.07 | 5906.18 | -1.987 | 1 | 1.926 | Yes |
| gga-miR-34c-3p | 110.455 | 564.405 | -1.217 | 0.909 | 2.353 | Yes |
| gga-miR-499-5p | 27.84 | 534.645 | -1.13 | 0.905 | 4.263 | Yes |
| gga-miR-6606-5p | 2.903 | 0.03 | 2.355 | 1 | -6.596 | Yes |
| gga-miR-7b | 39.625 | 0.03 | 3.785 | 1 | -10.367 | Yes |
| gga-miR-92-3p | 6410.387 | 14666.56 | -2.252 | 1 | 1.194 | Yes |
