# Supplementary material for: Chicken miR-148a-3p regulates immune responses against AIV by targeting the MAPK signalling pathway and IFN-γ
Source: Vet Res. 2023 Nov 22;54:110. doi: 10.1186/s13567-023-01240-3 (PMC10664352; doi:10.1186/s13567-023-01240-3)
Supplement: Supplementary file 2 — Additional file 2. List of immune-related target genes scoring over 80 in the miRDB. [file 13567_2023_1240_MOESM2_ESM.docx]

**Additional file 2**. **List of immune-related target genes which are over 80 score in miRDB database were showed.**

| **Target Score** | **Gene symbol** | **Gene name** | **Gene description** |
| --- | --- | --- | --- |
| 100 | ENSGALG00010019232 | SOS2 | SOS Ras/Rho guanine nucleotide exchange factor 2 [Source:HGNC Symbol;Acc:HGNC:11188] |
| 99 | ENSGALG00010018446 | FBN1 | fibrillin 1 [Source:HGNC Symbol;Acc:HGNC:3603] |
| 99 | ENSGALG00010011933 | IFNG | interferon gamma [Source:NCBI gene (formerly Entrezgene);Acc:396054] |
| 97 | ENSGALG00010021730 | ARL8B | ADP Ribosylation Factor Like GTPase 8B |
| 97 | ENSGALG00010019239 | CYTH3 | cytohesin 3 [Source:HGNC Symbol;Acc:HGNC:9504] |
| 96 | ENSGALG00010019470 | TGFB2 | transforming growth factor beta 2 [Source:NCBI gene (formerly Entrezgene);Acc:421352] |
| 95 | ENSGALG00010018396 | TBL1XR1 | transducin beta like 1 X-linked receptor 1 [Source:NCBI gene (formerly Entrezgene);Acc:426284] |
| 95 | ENSGALG00010004824 | ATM | ATM serine/threonine kinase [Source:NCBI gene (formerly Entrezgene);Acc:395401] |
| 93 | ENSGALG00010029184 | DYNLL2 | dynein light chain LC8-type 2 [Source:NCBI gene (formerly Entrezgene);Acc:417663] |
| 93 | ENSGALG00010015487 | RPS6KA6 | ribosomal protein S6 kinase A6 [Source:HGNC Symbol;Acc:HGNC:10435] |
| 92 | ENSGALG00010007719 | LTBP1 | latent transforming growth factor beta binding protein 1 [Source:NCBI gene (formerly Entrezgene);Acc:421461] |
| 90 | ENSGALG00010018317 | ZMAT3 | zinc finger matrin-type 3 [Source:HGNC Symbol;Acc:HGNC:29983] |
| 90 | ENSGALG00010018937 | INHBB | inhibin beta B subunit [Source:NCBI gene (formerly Entrezgene);Acc:396126] |
| 88 | ENSGALG00010013314 | KIT | KIT proto-onco, receptor tyrosine kinase [Source:NCBI gene (formerly Entrezgene);Acc:378783] |
| 88 | ENSGALG00010022667 | PTEN | phosphatase and tensin homolog [Source:HGNC Symbol;Acc:HGNC:9588] |
| 87 | ENSGALG00010001177 | SEH1L | SEH1 like nucleoporin [Source:HGNC Symbol;Acc:HGNC:30379] |
| 86 | ENSGALG00010005048 | ROCK1 | Rho-associated, coiled-coil containing protein kinase 1 [Source:NCBI gene (formerly Entrezgene);Acc:373970] |
| 84 | ENSGALG00010017553 | EDA | ectodysplasin A [Source:HGNC Symbol;Acc:HGNC:3157] |
| 84 | ENSGALG00010014707 | KPNA5 | karyopherin subunit alpha 5 [Source:HGNC Symbol;Acc:HGNC:6398] |
| 84 | ENSGALG00010001628 | MAPK11 | mitogen-activated protein kinase 11 [Source:NCBI gene (formerly Entrezgene);Acc:417739] |
| 84 | ENSGALG00010015876 | SKP1 | S-phase kinase-associated protein 1 [Source:NCBI gene (formerly Entrezgene);Acc:416319] |
| 83 | ENSGALG00010028015 | DUSP7 | dual specificity phosphatase 7 [Source:NCBI gene (formerly Entrezgene);Acc:415891] |
| 83 | ENSGALG00010019972 | PPP1CB | protein phosphatase 1 catalytic subunit beta [Source:NCBI gene (formerly Entrezgene);Acc:396019] |
| 83 | ENSGALG00010017035 | PIK3CD | phosphatidylinositol-4,5-bisphosphate 3-kinase catalytic subunit delta [Source:NCBI gene (formerly Entrezgene);Acc:419444] |
| 81 | ENSGALG00010001837 | TAB3 | TGF-beta activated kinase 1 (MAP3K7) binding protein 3 [Source:HGNC Symbol;Acc:HGNC:30681] |
| 81 | ENSGALG00010025512 | NRAS | neuroblastoma RAS viral onco homolog [Source:NCBI gene (formerly Entrezgene);Acc:419885] |
| 80 | ENSGALG00010027040 | CLIP1 | CAP-Gly domain containing linker protein 1 [Source:HGNC Symbol;Acc:HGNC:10461] |
