# Supplementary material for: Chicken miR-148a-3p regulates immune responses against AIV by targeting the MAPK signalling pathway and IFN-γ
Source: Vet Res. 2023 Nov 22;54:110. doi: 10.1186/s13567-023-01240-3 (PMC10664352; doi:10.1186/s13567-023-01240-3)
Supplement: Supplementary file 3 — Additional file 3. Sequences of primers used for quantitative PCR or cloning. [file 13567_2023_1240_MOESM3_ESM.docx]

**Additional file 3.** **Primer sequences used for RT-qPCR or cloning.**

| Genes |  | Primer sequence | Product  length (bp) | Annealing temp (°C) | | Assay | Reference |
| --- | --- | --- | --- | --- | --- | --- | --- |
| gga-mir-148a-3p* | F | UCAGUGCACUACAGAACUUUGU | - | 60 |  | | MIMAT0001120 |
|  | R | Universal Primer |  |  |  |  |  |
| U1A | F | CTGCATAATTTGTGGTAGTGG | - | 60 |  | | V00444 |
|  | R | Universal Primer |  |  |  |  |  |
| IFNGR2 | F | TGA GTG CAG TTT CCT CAA GT | 138 | 60 |  | | NM_001008676.3 |
|  | R | GGC CCT ATG GTA GTG TTT CT |  |  |  |  |  |
| OASL | F | TCA AGA CCG TCA AGG GCG | 199 | 60 |  | | NM_001397447.1 |
|  | R | GGA CTG GTG ATG CTG ACT CC |  |  |  |  |  |
| EIF2AK2 | F | TCA GTC TGA GTC ATG GGG TA | 148 | 50 |  | | NM_001397447.1 |
|  | R | AGG TGC CAA TAC TCT TCT GG |  |  |  |  |  |
| TNF-Α | F | CGCTCAGAACGACGTCAA | 114 | 60 |  | | XM_040694843.2 |
|  | R | TCGTCCCACACCAACGAG |  |  |  |  |  |
| TAB3 | F | CACCGCAAAGACCTGGGACTG | 81 | 60 |  | | XM_416787 |
|  | R | GTGGGTGCTGGTTTCGTTGAGATGGT |  |  |  |  |  |
| MAPK11 | F | TCC GCT AAA ATG TCC GAG C | 134 | 60 |  | | NM_001006227.1 |
|  | R | 5’- TCA TAA GCT GAA CAC ACG GA -3’ |  |  |  |  |  |
| IL6 | F | CTCGTCCGGAACAACCTCAA | 121 | 60 |  | | NM_204628 |
|  | R | AGGTCTGAAAGGCGAACAGG |  |  |  |  |  |
| TGF-β2 | F | AAGCTACCTGACTCCATTTG | 141 | 60 |  | | NM_001031045.3 |
|  | R | GCATTGCGATTCAAGTGTTA |  |  |  |  |  |
| Jun | F | CGC GGG CTC TGT TCT ATG | 118 | 55 |  | | NM_001031289 |
|  | R | TCA GCA CCT TGG CGT TAT TAT |  |  |  |  |  |
| DMB2 | F | CTGACGCTGGAGGTGACG | 97 | 55 |  | | NM_001135166 |
|  | R | GCCCGCCAGCAGTAGAC |  |  |  |  |  |
| IFN-β | F | CTTGCCCACAACAAGACGTG | 140 | 60 |  | | NM_001024836 |
|  | R | GTGTTTTGGAGTGTGTGGGC |  |  |  |  |  |
| TNF-α | F | CGCTCAGAACGACGTCAA | 115 | 60 |  | | XM_040694843.2 |
|  | R | TCGTCCCACACCAACGAG |  |  |  |  |  |
| MX1 | F | AGC CAT AGA ACA AGC CAG AA | 127 | 60 |  | | NM_204609.1 |
|  | R | GGT ACT GGT AAG GAA GGT GG |  |  |  |  |  |
| BF2 | F | CCATCCGGGGGTATTATCA | 105 | 60 |  | | NM_001031338.1 |
|  | R | TGGTGGGAACTGCCTCTG |  |  |  |  |  |
| IL-1β | F | TGGGCATCAAGGGCTACA | 244 | 60 |  | | NM_204524 |
|  | R | TCGGGTTGGTTGGTGATG |  |  |  |  |  |
| BLB2 | F | GCT CCA GGG ATG CTG AAT TA | 161 | 60 |  | | NM_001318995.2 |
|  | R | AGT CAG CGC GTT TAT TAG GA |  |  |  |  |  |
| IFN-γ | F | AACAACCTTCCTGATGGCGT | 106 | 60 |  | | [NM_205149.1](https://www.ncbi.nlm.nih.gov/entrez/viewer.fcgi?db=nucleotide&id=45433517) |
|  | R | TGAAGAGTTCATTCGCGGCT |  |  |  |  |  |
| GAPDH | F | TGCTGCCCAGAACATCATCC | 142 | 60 |  | | NM 204305 |
|  | R | ACGGCAGGTCAGGTCAACAA |  |  |  |  |  |
| WT-IFN-γ | F | CGAGCTCGACGTTGATACTGTACTCA | 169 | 60 | Luciferase Reporter Assay | | NM_205149.2 |
|  | R | CCAAGCTTGCTTCAGTGCATAACAATAAC |  |  |  |  |  |
| MT-IFN-γ | F  R | CGAGCTCGACGTTGATACTGTACTCA  CCAAGCTTGCTGACGGATATAACAATAAC | 169 | 60 | Luciferase Reporter Assay | | NM_205149.2 |
| WT-TGF-β2 | F | CGAGCTCCTTTTTCTCAAAATGCACTGAT | 179 | 60 | Luciferase Reporter Assay | | NM_001031045.4 |
|  | R | CCCAAGCTTTATAGAGGGGTGTTTCAC |  |  |  |  |  |
| MT-TGF-β2 | F | CGAGCTCCTTTTTCTCAAAAGTCATCGAT | 179 | 60 | Luciferase Reporter Assay | | NM_001031045.4 |
|  | R | CCCAAGCTTTATAGAGGGGTGTTTCAC |  |  |  |  |  |
| WT-MAPK11 | F | CGAGCTCTTCTGACAAAATTATGCACTGAG | 185 | 60 | Luciferase Reporter Assay | | NM_001006227.2 |
|  | R | CCCAAGCTTAGGATTGCTTGTATGGAATTC |  |  |  |  |  |
| MT-MAPK11 | F | CGAGCTCTTCTGACAAAATTAGTCATCGAG | 185 | 60 | Luciferase Reporter Assay | | NM_001006227.2 |
|  | R | CCCAAGCTTAGGATTGCTTGTATGGAATTC |  |  |  |  |  |

* Forward primer of miRNAs is designed to identical to the entire mature miRNA sequence and G was added to the 5’ end of the primer to adjust the Tm. Underlined sequences are restriction enzyme site used for cloning. The underlined letters represent the *Sac* I and *Hind* III enzyme loci for cloning: GAGCTC for *Sac* I and AAGCTT for *Hind* III.
