# Supplementary figures and images for: Chicken miR-148a-3p regulates immune responses against AIV by targeting the MAPK signalling pathway and IFN-γ
Source: Vet Res. 2023 Nov 22;54:110. doi: 10.1186/s13567-023-01240-3 (PMC10664352; doi:10.1186/s13567-023-01240-3)

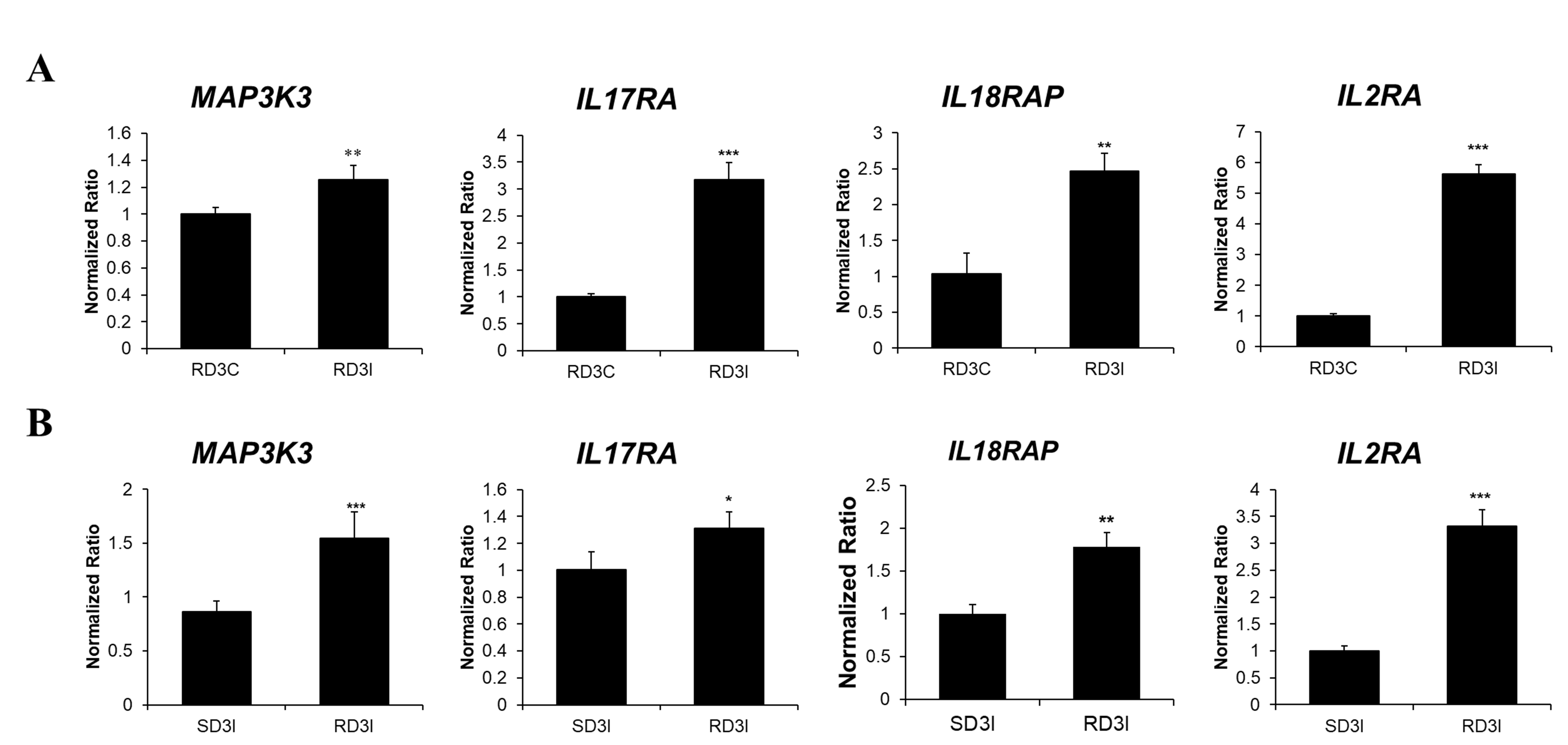

Supplement: Supplementary file 4 — Additional file 4. The gene expression of immune-related target genes (in the miRDB database) of gga-miR-148a-3p in the lung tissues of resistant chickens after H5N1 infection (A) and in H5N1-infected resistant chickens compared to H5N1-infected susceptible chickens (B). Expression levels were normalized to those of GAPDH and measured in triplicate. Significant differences in mRNA expression levels are indicated as follows: *, p < 0.05; **, p < 0.01; and ***, p < 0.001. Error bars indicate the SEM of technical replicates performed in triplicate. [file 13567_2023_1240_MOESM4_ESM.jpg]
